# The human gut metacommunity as a conceptual aid in the development of precision medicine

**DOI:** 10.3389/fmicb.2024.1469543

**Published:** 2024-10-10

**Authors:** Gerald W. Tannock

**Affiliations:** Department of Microbiology and Immunology, University of Otago, Dunedin, New Zealand

**Keywords:** metacommunity, microbiome, microbiota, precision medicine, symbiosis, dysbiosis

## Abstract

Human gut microbiomes (microbiotas) are highly individualistic in taxonomic composition but nevertheless are functionally similar. Thus, collectively, they comprise a “metacommunity.” In ecological terminology, the assembly of human gut microbiomes is influenced by four processes: selection, speciation, drift, and dispersal. As a result of fortuitous events associated with these processes, individual microbiomes are taxonomically “tailor-made” for each host. However, functionally they are “off-the-shelf” because of similar functional outputs resulting from metabolic redundancy developed in host-microbe symbiosis. Because of this, future microbiological and molecular studies of microbiomes should emphasize the metabolic interplay that drives the human gut metacommunity and that results in these similar functional outputs. This knowledge will support the development of remedies for specific functional dysbioses and hence provide practical examples of precision medicine.

## Introduction

The colon of humans is colonized by a microbial community, commonly known as the gut microbiome (microbiota), that is composed mostly of bacterial species (Franzosa et al., 2015; Parizadeh and Arrieta, 2023). The community has been studied in detail from the 1970s using feces as a proxy for colon samples, with particular emphasis on taxonomic composition (“who is there?”) for the last 25 years. This became possible because of the availability and development of high throughput sequencing of bulk DNA extracted from feces, and subsequent sequence analysis (Franzosa et al., 2015; Parks et al., 2017; Asnicar et al., 2024; Parizadeh and Arrieta, 2023). In general, the community contains about twenty trillion bacterial cells in which members of the Bacillota (Firmicutes) and Bacteroidota (Bacteroidetes) form about 85% of the microbiome. Three bacterial families, the *Lachnospiraceae*, *Ruminococcaceae*, and *Bacteroidaceae* are well represented (White et al., 2014; Sender et al., 2016; Rampelli et al., 2020). Much of the research interest in the gut microbiome has focussed on defining the taxonomic composition of the “normal” or “healthy” microbiome but this is an impossible goal due to the huge variance in microbiome compositional diversity between individual humans at genus, species, and strain levels (Turnbaugh et al., 2009; Ursell et al., 2012; Shanahan et al., 2021). This article proposes that viewing the gut microbiome as a metacommunity which has functional consistency will aid the development of precision (personalized; individual) medicinal interventions to restore health.

## What is a metacommunity?

Communities are interactive assemblages of species that are characteristic of a specific habitat or ecosystem. The formation of communities is influenced by four processes: environmental selection (deterministic fitness differences among species), ecological drift (stochastic changes in species abundance), local diversification (creation of new species), and dispersal (spatial movement of species) (Vellend, 2010). These processes have been studied in relation to the human gut microbiome and observations that are pertinent to this article follow.

1. Selection of bacterial species that have biochemical fitness determinants appropriate for catabolism of dietary components and host secretions is apparent among the members of the gut microbiome. Key indicators of selection are genetic features such as Polysaccharide Utilization Loci (PULs) that encode binding proteins, hydrolytic enzymes (carbohydrate-active enzymes), and transport proteins associated with the bacterial cell surface. Products of PULs sequester and degrade plant-derived glycans that are common in human food (for example, resistant starch, hemicelluloses such as complex xylans, and pectins) (El Kaoutari et al., 2013; Grondin et al., 2017). At least some of these specialized bacteria are keystone species that initiate the catabolism of complex carbohydrates that subsequently fuel the metabolism of consortia (guilds) (Ze et al., 2012).
2. Temporal drift in climax communities of individuals has been studied and, in general, the abundances of species is relatively constant over time (Faith et al., 2013). However, the influences of allochthonous factors such as dietary fiber, medications and environment are apparent (David et al., 2014; Tannock, 2021a; Nagata et al., 2022; Gacesa et al., 2022). The microbiomes of infants and children follow characteristic colonization patterns that are mostly influenced by trophic factors and are linked to dispersal of species (see below). The direct and indirect impact of endogenous factors such as predation by bacteriophages on species abundance has been measured in gnotobiotic mouse experiments and may have a modulating effect on some bacterial populations (Hsu et al., 2019). The ecological impact of bacteriocins and other antimicrobial substances encoded by biosynthetic gene clusters (BGC) is unknown (Tannock, 2022).
3. Speciation can best be understood by considering the widespread presence of bacterial strains (subsets of species) in the gut. Mutation of genes occurs commonly; *de novo* mutations are estimated at 2 × 10^9^ to 6 × 10^12^ single nucleotide polymorphisms per microbiota per day. Gene loss and gene gain (by horizontal gene transfer) in bacterial species reveals a genetically mutable community in real time (Nielsen et al., 2014; Zhao et al., 2019). Consequently, strains of the same species differ in genetic characteristics where “core” genes common to all strains plus “dispensable” genes (variable presence) together comprising the pangenome, can be recognized (Medini et al., 2005; Truong et al., 2017).
4. Dispersal refers to the movement (transmission) of species that establish or augment communities in other sites. In terms of the human gut microbiome, the gut of each new-born infant offers a pristine environment for colonization by bacterial species. The maternal fecal microbiome is a major source of bacterial strains during the first few months of life on the assemblage of the “new” community. Although vertical transmission is very important in this dispersal, horizontal transmission from paternal, family and environmental sources also occurs (Song et al., 2013; Gaulke and Sharpton, 2018; Yassour et al., 2018; Tannock, 2021b; Gacesa et al., 2022; Valles-Colomer et al., 2023; Dubois et al., 2024). A community of low diversity emerges to begin with that, while infants are suckled at the breast, is dominated by species that can catabolize human milk components including human milk oligosaccharides (Mills et al., 2023). After weaning, this markedly trophic colonization is replaced by a more neutral (stochastic) model that is nevertheless driven by niche differentiation whereby bacterial species that catabolize dietary fiber and host secretions form the nucleus of a community that, especially in terms of emergent properties, eventually resembles that of adults in general (Leong et al., 2018; Lawley et al., 2019).

**Figure 1:**
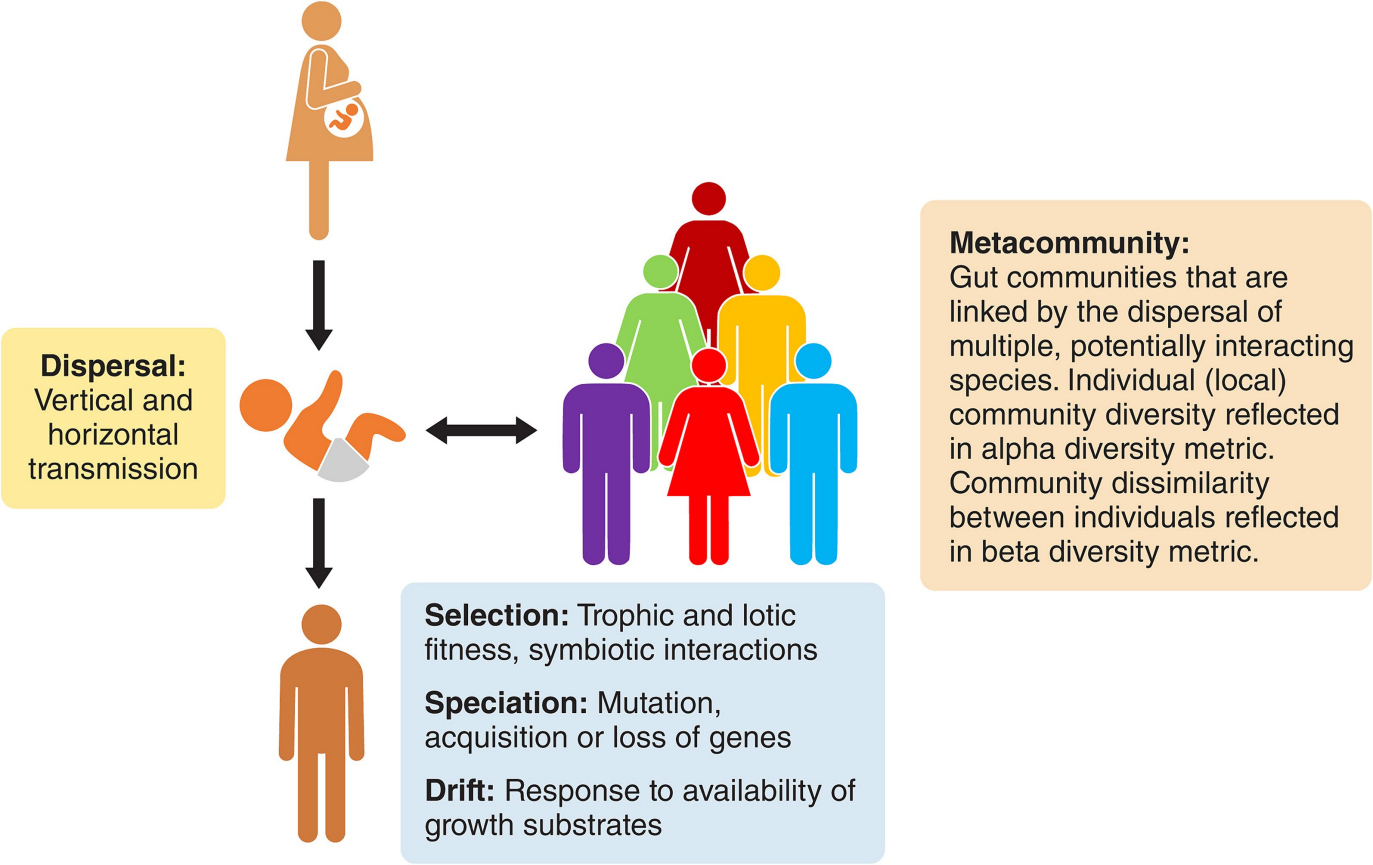
The gut microbiome of humans represented as a metacommunity. Each human is inhabited by an ecological community that is different from that of other humans, but the communities are nevertheless linked by dispersal and modified by drift, speciation, and selection. They have similar emergent properties due to metabolic redundancy among the otherwise disparate bacterial constituents.

As a result of these processes, each person’s gut microbiome is like that of an island within an archipelago with a microbial assemblage that is individualistic with regards to taxonomic composition (Costello et al., 2012). These personal climax communities can be viewed as subsets of a “metacommunity” that are linked by the potential or actual dispersal of interacting species (Figure 1). Indeed, the dispersal of members of human microbiomes is not limited to early life. In work targeting 7,646 fecal samples from multinational sources, Valles-Colomer et al. (2023) provided strain-level metagenomic evidence of microbiome dispersal between adults who shared environments (strain sharing rate about 12%). Greater proportions of shared strains were detected in oral microbiomes than in the case of the gut, but the data nevertheless support the view that the human gut microbiome is a metacommunity whose subsets are linked by potential dispersal and acquisition throughout life.

Despite variation in taxonomic composition, metacommunity islands have common functional attributes derived from symbiotic relationships between microbiome and host that have developed during co-evolution (Tannock, 2024a). This is particularly apparent through study of the interaction of species in food webs. Community food webs in the gut are interlocking and interdependent food chains that originate in the catabolism of complex dietary components (plant glycans) and host secretions (bile acids, mucins) and develop through cross feeding of simple carbohydrates, amino acids, vitamins, and organic acids (e.g., formate, fumarate, succinate, lactate, succinate) between species (Lindstad et al., 2021; Culp and Goodman, 2023). These trophic interactions are consistent with the concept of the provision of “public good services” by some species for collective benefit of the community (Morris et al., 2012).

Food webs, like pathways in cellular processes, are nonlinear networks that are controlled by feedback loops. “Interspecies hydrogen transfer” is a good example of a feedback loop in the gut. Hydrogen is produced during fermentations, but the environmental concentration is kept low in the gut ecosystem by hydrogen consumers using acetogenesis, dissimilatory sulfate reduction, hydrogenotrophic respiration, or methanogenesis. Fermentative bacteria can live in the colon at the limits of what is thermodynamically possible because NADH oxidation can be coupled to proton reduction under these low hydrogen concentrations (Stams and Plugge, 2009).

Some metabolic functions within networks can be achieved by different biochemical pathways, hence different species may carry out the same function (metabolic redundancy, such as in butyrate production) (Pryde et al., 2002; Tian et al., 2020). Unrelated humans share 82% of bacterial metabolic pathways detected in fecal DNA but only 43% of bacterial species (Visconti et al., 2019). This helps to explain the taxonomic individualism, but similar metabolic outputs, of human gut microbiomes.

## Measuring the “health” of the metacommunity

Most members of human gut microbiomes are obligate anaerobes that die within a short time under aerobic conditions, and many are autotrophs that require cross-feeding of nutrients from other community members (Tramontano et al., 2018; Nayfach et al., 2019). The logistical and technical difficulties of conducting large scale, culture-based analysis under these circumstances led to the adoption of metagenomic, phylogenetic comparisons of fecal microbiomes. Some studies using this technology indicated that the relative abundances of certain bacterial groups in fecal microbiomes indicated health or disease (Manor et al., 2020). Whether these differences were causative of disease or collateral damage due to disease could not be established by these observational studies. Relatively few human participants were sampled and there are numerous confounding factors that render it difficult to reproduce the results from one study to another (Sze and Schloss, 2016). More recent studies, using machine learning procedures, use data from larger groups of people and focus on bacterial strain differences (Manor et al., 2020). However, repeatability of these studies is also untested, and they sometimes seem to be inventories of microbial diversity rather than critical testing of hypotheses (Prosser, 2022).

An alternative school of thought in relation to defining a healthy microbiome has recently been published together with an overall framework for future developmental research (Tannock, 2024b). In brief, the proposal states that bioassays should be developed to measure the functioning of microbiomes. This would be analogous to the use of “lab tests” of peripheral blood, widely used in medical diagnostics. A range of metacommunity “normal values” would first be established to which values from individual microbiomes would be compared. Potential bioassays to assess the health of the symbiosis between gut microbiome and human host include measuring undegraded plant glycans in fecal samples, fecal SCFA profiles, fecal bile acid profiles (for example, proportion of secondary bile acids), fecal mucin profiles, fecal agonists of G protein-coupled receptors (for example, short chain fatty acids and *N*-acyl amides), plasma/serum metabolomes, qPCR quantitation of bacterial genetic loci known to underpin catabolic features of symbiosis (for example, genes encoding carbohydrate-active enzymes [CAZymes]), and immune factors present in feces (for example, amounts of secretory IgA and calprotectin). It is proposed that these kinds of assays, informed by knowledge of community function, are likely to be more useful than taxonomic comparisons in differentiating healthy microbiotas from unhealthy because functional outputs of microbiomes are similar across the metacommunity of healthy humans.

## Current research gaps in understanding the gut metacommunity

As mentioned previously, changes to gut community ecology may contribute to the increased prevalence of metabolic conditions (for example, obesity, cardiovascular disease, type 2 diabetes) in humans living in, or adopting, industrialized lifestyles. Altered functioning of the microbiome and hence altered host-microbe equilibrium (“dysbiosis”) might be involved. If that is correct, remedial action might be possible.

Restoration in westerners of “missing microbes” cultured from people following non-industrialized lifestyles has been suggested but would probably not succeed because the diet of recipients is unlike that of the donors, so niches for them in the gut are lacking (De Filippo et al., 2010). It would take some strong arguments to persuade people in industrialized countries to make transitions to ancestral diets and lifestyle (Burger et al., 2012). Modulation of gut microbiome function of humans by less dramatic dietary intervention is realistic (Tannock, 2021a), but there needs to be much better evaluation of habitual diets of humans participating in microbiome trials (Renall et al., 2023). Ethnicity of human participants also needs to be recorded because dietary preferences and lifestyles may be influenced (Gaulke and Sharpton, 2018; Xu et al., 2020). Control human cohorts in gut microbiota studies are frequently described as “healthy” but anthropometric tests that show this to be valid, rather than relying on personal perceptions, should be used to confirm health status (Renall et al., 2023).

Gut transit time should be measured in every study of the gut microbiome because it is variable between humans and is influenced by the amount of dietary fiber that is consumed. Community function is influenced by transit time because slower passage of digesta through the colon allows time for catabolism of most dietary carbohydrates in the proximal colon. Bacterial metabolism in the distal colon then turns to the use of amino acids as substrates with the generation of branched SCFAs (isobutyrate, isovalerate). Thus, the metabolic profile of the community can vary according to temporal loading of nutrients in colonic regions (Roager et al., 2016; Vandeputte et al., 2016; Asnicar et al., 2021).

The gut metacommunity of humans continues to evolve. This is revealed by comparison of fecal microbiomes in samples collected from people who are nomadic hunter-gatherers in Africa, agrarian populations living in countries where industrialization is minimal, in developed countries that are highly industrialized, and people who are in transition (migrants) between non-industrialized and industrialized regions or countries (De Filippo et al., 2010; Vangay et al., 2018; Schnorr et al., 2014; Shanahan et al., 2022; Tamburini et al., 2022; Blanco-Míguez et al., 2023; Carter et al., 2023). In general, industrialization selects for gut communities of lower diversity due to consumption of diets containing more refined grains, and less coarse dietary fiber (De Filippo et al., 2010). More studies on the microbiomes of non-western societies are required because they may reveal further molecular specializations of gut bacteria that are critical to catabolism of plant glycans. These studies will also define the “normal values” of ecosystem function for Asian and other societies.

Curing dysbiosis means that restoration of the gut ecosystem must occur. To do this, we need to know what the ecosystem was like, especially how it functioned, when it was healthy (Wainwright et al., 2018). For example, we might gain an appreciation of ecosystem function by focussing on nutritional features, such as cross-feeding, that are essential for promoting community diversity (Germerodt et al., 2016). There is a need to continue to investigate the ecological importance of spatial heterogeneity in the colon, especially the possibility that there are multiple habitats associated with complex plant glycan molecules (Pereira and Berry, 2017; Tannock and Taylor, 2017).

**Figure 2:**
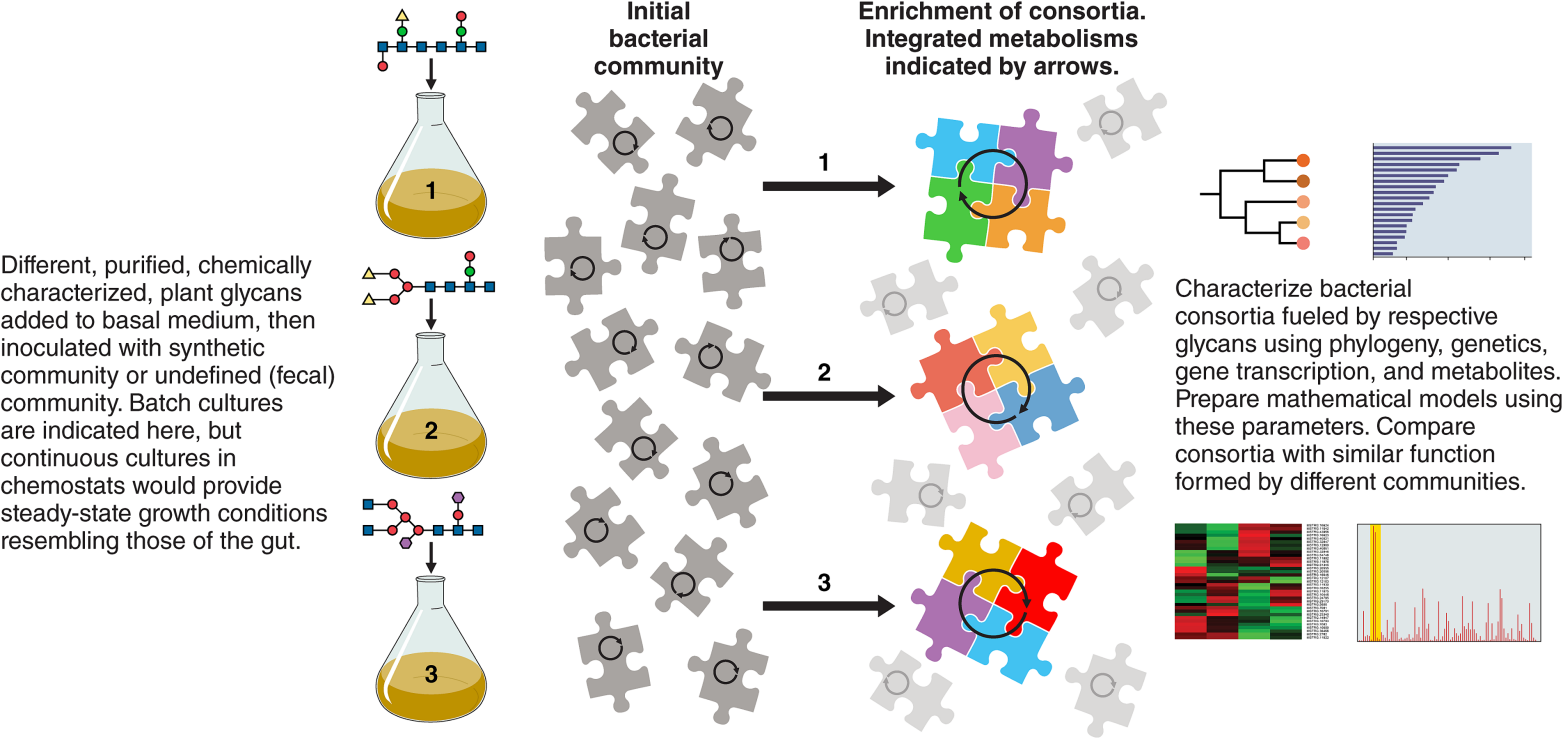
Research with cultured, defined or non-defined bacterial communities fed plant glycans and characterized phylogenetically, genetically, transcriptionally, and metabolically are suggested as starting points to reveal the consortia that drive the energetics of the community in relation to specific growth substrates. These experiments could identify keystone species and explain the individualism of gut microbiomes which are nevertheless similar in emergent properties because of metabolic redundancy. Potentially useful information about bacterial growth substrates for use in ecosystem restoration could be gained.

As summarized in Figure 2, the use of culture-based experiments with “synthetic” microbial communities may be advantageous in future research because they could model specific functions occurring in the gut ecosystem that can in turn be modulated by interventions (Widder et al., 2016; Brüls et al., 2021). These model, *in vitro* communities could have simplified bacterial diversity yet perform the complex functions observed *in vivo* and could be investigated by gene transcription and biochemistry to generate regulatory information (“how does it work?”) especially in relation to temporal nutrient switching, which may be an important feature of bacterial residency in the colon (Centanni et al., 2020a). Enrichment cultures, from which models could be derived using medium containing a specific plant glycan and a fecal inoculum, could be useful because metabolically cohesive, bacterial consortia, about which we know little, would be enriched and the rules that drive their formation would be revealed (Pascual-García et al., 2020). Work with co-cultures, preferably performed under lotic conditions such as in chemostats (steady-state conditions), provide opportunities for replication of experiments and hence improve reproducibility and statistical confidence, and potential for mathematical modeling (Kettle et al., 2015; Zomorrodi and Segrè, 2016; Centanni et al., 2019; Centanni et al., 2020b; Liu et al., 2020; Mabwi et al., 2020; Sims and Tannock, 2020; Shetty et al., 2022; Gianetto-Hill et al., 2023). Mutation of specific genes can test ecological fitness of bacteria showing the importance of specific bacterial attributes in underpinning symbiont life in the gut (Sims et al., 2011; Wilson et al., 2012; Tannock et al., 2012; Wilson et al., 2014). Overall, studying molecular details of the metacommunity should reveal intriguing details about how it came to be the way it is today, how its “health” can be better measured, and how it might be remediated for medical purposes.

## Potential future developments to restore microbiome function using precision medicine

Personal gut microbiomes, collectively, form a conceptual metacommunity that has functional criteria consistent with “health” of the microbial community. The taxonomic composition of personal microbiomes is “tailor-made” by fortuitous events associated with selection, drift, speciation, diversification, and dispersal. However, ecological functions are similar across the metacommunity due to selection of metabolic redundancies, so functionality is “off-the-shelf” regardless of taxonomic composition. This means that restorative measures to achieve health can avoid the difficulties associated with altering the taxonomic composition of the microbiome (which is vastly different between individual humans) and can focus instead on correction of specific functional differences. However, an understanding of how the metacommunity “works” is required to develop practical solutions.

The idea that health care can be tailored according to the patient’s genotype, environment and lifestyle rather than the expected responses of an “average patient” underpins the concept of “precision” (“personalised,” “individualised”) medicine (Kuntz and Gilbert, 2017; Petrosino, 2018; McCormick and Chang, 2021). Applied to the gut microbiome, the aim would be to remediate dysbiosis revealed by bioassays of microbiome functions using personalized treatments, rather than traditional probiotic and/or prebiotic approaches based on merchandising “wellness.” Precision medical approaches are feasible because responses to dietary supplementation with plant glycans (for example, response to doses of arabinoxylan or resistant starch) are variable among humans (Martínez et al., 2010; Nguyen et al., 2020; Holmes et al., 2022; Lancaster et al., 2022) thus providing possibilities of personalized nutrition. The scope of this kind of work has expanded recently because of better analytical methods to determine and modify glycan chemistry (Amicucci et al., 2019; Michalak et al., 2020; Tuncil et al., 2020; Deehan et al., 2020; Cantu-Jungles et al., 2021; Castillo et al., 2022; Couture et al., 2024), coupled with ever expanding knowledge of the biochemical diversity of plant cultivars, as well as an interest in using processed plant wastes to prepare novel foods for human consumption (Delannoy-Bruno et al., 2021).

Admittedly, a dysbiotic gut community might lack the microbial mechanisms that drive normal functions (for example, catabolism of resistant starch) (Walker et al., 2011). There could, therefore, be a need to prepare commercial, multi-component bacterial consortia with prescribed functional attributes and to transfer the artificial community to specific humans. This might be accomplished by a dose of a “defined function consortium” administered orally or by enema. This is analogous to restoring a disturbed colon community by means of “faecal microbiota transplant” (FMT) which is useful in treating some cases of *Clostridioides difficile* infection and may also be useful in ameliorating disrupted transfer of gut bacteria to children that have been delivered by cesarean section. However, the biology of FMT is ill-defined and not free of medical risk so inoculation with laboratory assembled consortia would be a sensible development (Gilbert and Lynch, 2019; Burke and Lamont, 2013; Smillie et al., 2018). Much work is required to not only develop pertinent consortia to achieve this goal, but also to prepare preparations that retain viability for use as inoculants, as well as details of dose and dosing schedule. Although the microbiome may be more malleable temporally than previously thought (Valles-Colomer et al., 2023), colonization resistance may be a limiting factor.

Considerations of the restoration of dysbiotic microbiomes based on knowledge of the human gut metacommunity has implications in another area of precision medicine, that of responses to cancer therapy. Receptor-ligand interactions (for example, PD-1, PD-L1) are associated with the ability of T-cells to differentiate between healthy human cells and potential pathogens. Unfortunately, cancer cells can co-opt this “immune checkpoint” system to avoid the destructive attentions of T-cells. Immunoglobulin administration is used to enhance therapy of several types of cancer by interfering with this immune checkpoint blockade (ICB). Destruction of cancer cells by T-cells is enhanced, although healthy cells are also affected. Curiously, not all patients respond equally to ICB treatment. Prior treatment with antibiotics reduces efficacy, indicating an influence of the gut microbiome. Research results using germfree and gnotobiotic mice suggest that some members of the microbiome promote response to ICB but this work is difficult to reconcile with human patients because the murine gut microbiome is dominated by different taxa and is differently distributed in the gut compared to humans (Cammarota et al., 2020; Fernandes et al., 2022; Park et al., 2023). Nevertheless, precision modulation of the human gut microbiome of cancer patients to enhance ICB could be useful spin-off technology from development of functional adjustments to restore healthy microbiomes.

Clearly, the starting point for future research is the development of bioassays (such as those outlined above) and others (such as catabolism of resistant starch and hemicelluloses) (Walker et al., 2011), by which health standards can be set in relation to the human gut metacommunity. Functional dysbiosis will then be recognizable in personal microbiomes of patients, and restorative procedures could be developed. “Precision functional restoration” will be the goal of this exciting research focussing on the human-microbiome symbiosis.

## Data Availability

The original contributions presented in the study are included in the article/supplementary material, further inquiries can be directed to the corresponding author.
